# Loss of endosomal recycling factor RAB11 coupled with complex regulation of MAPK/ERK/AKT signaling in postmortem spinal cord specimens of sporadic amyotrophic lateral sclerosis patients

**DOI:** 10.1186/s13041-019-0475-y

**Published:** 2019-06-13

**Authors:** Joy Mitra, Pavana M. Hegde, Muralidhar L. Hegde

**Affiliations:** 1 Department of Radiation Oncology, Houston Methodist Research Institute, Houston, TX 77030 USA; 2 Weill Medical College, New York, NY 10065 USA; 3 Houston Methodist Neurological Institute, Institute of Academic Medicine, Houston Methodist, Houston, TX 77030 USA

**Keywords:** ALS, RAB11, AKT/MAPK/ERK pathways, Synaptic dysfunction, TDP-43

## Abstract

Synaptic abnormalities, perturbed endosomal recycling mediated by loss of the small GTPase RAB11, and neuroinflammatory signaling have been associated with multiple neurodegenerative diseases including the motor neuron disease, amyotrophic lateral sclerosis (ALS). This is consistent with the neuroprotective effect of RAB11 overexpression as well as of anti-inflammatory compounds. However, most studies were in animal models, and this phenomenon has not been demonstrated in human patients. Moreover, crosstalk between endosomal trafficking and inflammatory signaling pathways in ALS remains enigmatic. Here, we investigated RAB11 expression and MAPK/ERK/AKT signaling in 10 post-mortem spinal cord specimens from patients with sporadic ALS and age-matched controls. All 10 ALS patients showed TDP-43 pathology, whereas two specimens showed an overlapping FUS pathology and one had an acquired Q331K mutation in TDP-43. There was consistent RAB11 downregulation in all ALS cases, while p-AKT and phospho-ribosomal S6 kinase (p-p90RSK) were upregulated. Furthermore, competition between AKT and ERK pathways was observed in ALS, suggesting subtle differences among the TDP-43-ALS subtypes, which may influence patient therapeutic responses. Our findings demonstrate a complex regulation/perturbation pattern of signaling cascades involving MAPK/AKT/RAB11 in spinal cord tissue from ALS patients. These results underscore the relationships between ALS pathology, altered neuronal trafficking, and inflammation.

**Electronic supplementary material:**

The online version of this article (10.1186/s13041-019-0475-y) contains supplementary material, which is available to authorized users.

## Main text

Amyotrophic lateral sclerosis (ALS) is a fatal degenerative disorder of motor neurons. The major subtype of ALS (~ 97% of cases) is associated with TAR DNA binding protein of 43 kDa (TDP-43) proteinopathy characterized by nucleo-cytosolic mislocalization [1]. Synaptic dysfunction and loss of vesicular trafficking have emerged as vital early factors in the etiologies of neurodegenerative diseases involving protein aggregates, which may develop decades before overt motor symptoms [2]. The endosomal recycling factor RAB11 (Ras-related protein) is a critical member of the Rab family; these small GTPases act as master-regulators for axonal transport of neurotrophin receptors and β1 integrins in dorsal root ganglion neurons, which is essential for their development, survival, and functionality [3]. Rab GTPase dysregulation has been consistently linked to defective vesicular trafficking, endosomal recycling, and autophagy in neurodegeneration [4]. Recent studies identified inhibition of endosomal trafficking due to loss of TDP-43 [5] and these defects were salvaged by RAB11 expression [6]. Furthermore, RAB11 signaling may crosstalk with essential immune-signaling pathways like mitogen-activated protein kinases/extracellular signal-regulated kinase 1 and 2 (MAPK/ERK1/2), as well as modulate AKT (protein kinase B)-mediated neuroinflammation [7]. Although initial activation by their respective tyrosine/serine phosphorylation is protective, persistent activation eventually promotes apoptosis.

**Fig. 1:**
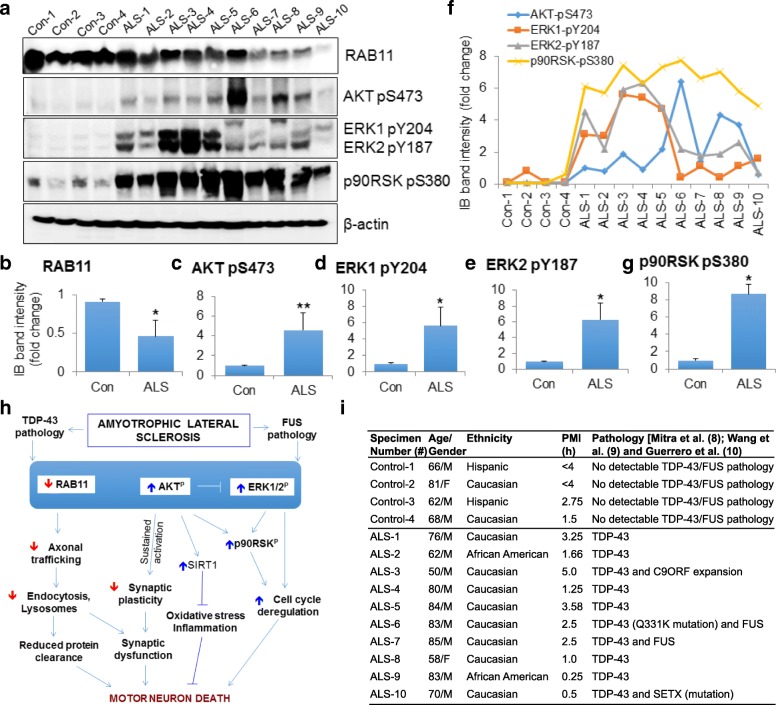
Complex regulation of RAB11 and AKT/MAPK/ERK signaling in ALS. **a** Total spinal cord (postmortem) tissue extracts from four controls and 10 ALS patients were immunoblotted using a pathway-specific antibody cocktail (Abcam# ab151279). **b-g** Protein levels were quantitated by band intensity measurements, and mean ± SD values were plotted as histograms. *, *p* < 0.1; **, *p* < 0.05. **f** Plot representing correlations between p-AKT and p-ERK1/2 levels in ALS. **h** Model showing dynamic and complex activation of RAB11, AKT, and ERK signaling in ALS subtypes. Loss of RAB11 may lead to defective axonal trafficking and perturbed endosomal recycling, both of which may contribute to synaptic abnormalities. While activation of AKT signaling is protective, sustained activation may contribute to synaptic dysfunction and oxidative stress-mediated neuroinflammation. The competitive nature of AKT versus ERK signaling may contribute to underlying disease processes and influence patient response to therapeutics. **i** Clinical features of control and ALS patients

In this report, we investigated the correlation between RAB11 loss and AKT/ERK signaling in post-mortem spinal cord tissue from patients with sporadic ALS. Ten ALS and four age-matched control samples were obtained from the Department of Veterans Affairs Brain Biorepository (USA). As tabulated in Fig. 1i and documented in our recent publication [8], all 10 ALS specimens showed strong TDP-43 pathology, whereas two (#6 and #7) exhibited overlapping TDP-43 and FUS (another RNA/DNA-binding ALS protein) pathology [9]. ALS#6 also harbored a Q331K mutation in the *TARDBP* gene that encodes TDP-43 [10]. We recently reported that loss of functional TDP-43 and FUS in these patients is linked to genome damage accumulation and apoptosis due to defective DNA strand-break repair [8, 9].

Here, we performed immunoblotting of total protein extracts isolated from cervical spinal cord tissues using the pathway-specific antibody cocktail (Abcam, Cat#ab151279) to assess RAB11 levels and its relationship with AKT/ERK signaling. RAB11 protein levels were decreased in all ALS cases [at least 2-fold on average, mean ± standard deviation (SD)] compared with controls (Figs. 1a and b). Interestingly, in majority of ALS specimens with higher loss of monomeric TDP-43 and more aggregation (ALS #9 and #10) [8] showed significantly reduced RAB11 levels (Additional file 1: Figure S1), suggesting that impaired RAB11-mediated protein clearance may contribute to TDP-43 toxicity. However, the inconsistency in direct correlation between TDP-43 proteinopathy and loss of RAB11 in some cases may be due to their complex crosstalk and underlying secondary pathology, for example, Senataxin (SETX) (chr9:135202108 T > C) pathology in ALS#10 and C9ORF extension in ALS#3. AKT activation by phosphorylation at serine 473 (p-AKT) is critical for synaptic function and managing oxidative stress-associated neuroinflammation. TDP-43-ALS cases showed ~ 4-fold (mean ± SD) higher levels of p-AKT (Figs. 1a, and c), which was distinct from the mutant SOD1-ALS subtype [11]. Notably, the highest p-AKT level (≥6-fold) was observed in the ALS#6 spinal cord bearing a sporadic mutation of Q331K in TDP-43 that was associated with increased TDP-43 fragmentation along with overlapping FUS pathology [9, 10]. Furthermore, the activated forms of ERK1 (phosphorylated at tyrosine 204) and ERK2 (phosphorylated at tyrosine 187) were ~ 6-fold (mean ± SD) higher in ALS cases compared with controls (Figs. 1a, d and e). The p-AKT levels inversely correlated with p-ERK1/2 in ALS, although both were higher compared with controls (Fig. 1f). This was most evident in ALS#3, #4, and #5 versus #6 and #8. It is also consistent with previous studies that show a negative regulation between AKT and ERK signaling pathways, even though they have a common downstream factor p90 ribosomal S6 kinase (p90RSK) [12, 13]. Hyperactivation of ERK1/2 signaling has been shown to contribute to neuronal cell cycle re-entry [14, 15]. Based on this, we speculate that such a phenotype may develop in subset of ALS patients with predominant ERK pathway activation, which may be addressed in future study. Independent of the extent of AKT or ERK1/2 signaling, p90RSK phosphorylation (serine 380) was significantly higher (≥8-fold, mean ± SD) in all ALS cases (Figs. 1a and g). Furthermore, although overall fold-changes of p-AKT and p-p90RSK proteins were higher in ALS patients compared with controls, ALS#6 patient with a TDP-43 Q331K mutation had the highest level of p-AKT and downstream factor p-p90RSK but comparable levels of p-ERK1/2. The total AKT and ERK1/2 levels were comparable in ALS specimens or controls (Additional file 2: Figure S2).

The inverse correlation between p-AKT and p-ERK1/2 levels is likely dependent on the underlying pathology, including TDP-43 aggregation versus fragmentation phenotype and/or its overlap with either FUS, C9ORF72 or SETX pathology. This reveals the complexity of the disease mechanisms and underscores the dynamic crosstalk involving direct or inverse relationships between these pathways.

In conclusion, our study shows overall dysfunction in a complex network of signaling cascades involving MAPK/AKT/RAB11 pathways associated with neurodegeneration in ALS (summarized in Fig. 1h). As highlighted in our comprehensive review of complexity in ALS subtypes [1], there are substantial differences in the underlying pathologies of a dozen ALS subtypes. Consistently, our results emphasize subtle differences and the complex regulation among signaling pathways in TDP-43 and, C9ORF72-, SETX- or FUS-associated overlapping ALS pathologies, which may provide clue on enigmatic ALS disease mechanisms for further investigation and therapeutic considerations.

## Additional files

Additional file 1: **Figure S1**. Relative levels of monomeric TDP-43 and RAB11 in ALS patients spinal cord tissue. Related to Fig. 1a. Histogram plot showing relative expression levels of monomeric TDP-43 and RAB11 in spinal cord tissue extracts from four controls and 10 ALS patients. X-axis indicates sample number and Y-axis denotes relative protein level, quantified from western blots. The TDP-43 monomer levels were previously reported in Mitra et al. [8] and RAB11 levels are shown in Fig. 1a in this study. (PDF 481 kb)

Additional file 2: **Figure S2**. AKT/MAPK/ERK signaling in TDP-43-ALS patients. Related to Fig. 1a. Representative western blot images of total spinal cord (post-mortem) tissue lysates from four controls and 10 ALS probed with anti-ERK1 (GTX100699), anti-ERK2 (GTX113094), anti-p90RSK (sc-74459), anti-AKT (sc-271149) and anti-GAPDH (NB-300-285) antibodies. GAPDH served as the loading control. (PDF 484 kb)

## Data Availability

All data generated or analyzed during this study are included in this published article.

## Abbreviations

AKT: Protein kinase B (PKB)
ALS: Amyotrophic lateral sclerosis
ERK: Extracellular signal-regulated kinase
MAPK: Mitogen-activated protein kinase
RAB11: Ras-related protein RAB11
RSK: Ribosomal S6 kinase

## References

[1] Guerrero EN, Wang H, Mitra J, Hegde PM, Stowell SE, Liachko NF, Kraemer BC, Garruto RM, Rao KS, Hegde ML (2016). TDP-43/FUS in motor neuron disease: complexity and challenges. Prog Neurobiol.

[2] Milnerwood AJ, Raymond LA (2010). Early synaptic pathophysiology in neurodegeneration: insights from Huntington's disease. Trends Neurosci.

[3] Bucci C, Alifano P, Cogli L (2014). The role of Rab proteins in neuronal cells and in the trafficking of neurotrophin receptors. Membranes (Basel).

[4] Kiral FR, Kohrs FE, Jin EJ, Hiesinger PR (2018). Rab GTPases and membrane trafficking in neurodegeneration. Curr Biol.

[5] Schwenk BM, Hartmann H, Serdaroglu A, Schludi MH, Hornburg D, Meissner F, Orozco D, Colombo A, Tahirovic S, Michaelsen M (2016). TDP-43 loss of function inhibits endosomal trafficking and alters trophic signaling in neurons. EMBO J.

[6] Deshpande M, Feiger Z, Shilton AK, Luo CC, Silverman E, Rodal AA (2016). Role of BMP receptor traffic in synaptic growth defects in an ALS model. Mol Biol Cell.

[7] Winter JN, Jefferson LS, Kimball SR (2011). ERK and Akt signaling pathways function through parallel mechanisms to promote mTORC1 signaling. Am J Physiol Cell Physiol.

[8] Mitra J, Guerrero EN, Hegde PM, Liachko NF, Wang H, Vasquez V, Gao J, Pandey A, Taylor JP, Kraemer BC, Wu P, Boldogh I, Garruto R, Mitra S, Rao KS, Hegde ML (2019). Motor neuron disease-associated loss of nuclear TDP-43 is linked to DNA double-Strand break repair defects. Proc Natl Acad Sci U S A.

[9] Wang H, Guo W, Mitra J, Hegde PM, Vandoorne T, Eckelmann BJ, Mitra S, Tomkinson AE, Van Den Bosch L, Hegde ML (2018). Mutant FUS causes DNA ligation defects to inhibit oxidative damage repair in amyotrophic lateral sclerosis. Nat Commun.

[10] Guerrero EN, Mitra J, Wang H, Rangaswamy S, Hegde PM, Basu P, Rao KS, Hegde ML. Amyotrophic lateral sclerosis (ALS)-associated TDP-43 mutation Q331K prevents nuclear translocation of XRCC4-DNA ligase 4 complex and is linked to genome damage-mediated neuronal apoptosis. Hum Mol Genetics. 2019. 10.1093/hmg/ddz062. [Epub ahead of print].

[11] Peviani M, Cheroni C, Troglio F, Quarto M, Pelicci G, Bendotti C (2007). Lack of changes in the PI3K/AKT survival pathway in the spinal cord motor neurons of a mouse model of familial amyotrophic lateral sclerosis. Mol Cell Neurosci.

[12] Hayashi H, Tsuchiya Y, Nakayama K, Satoh T, Nishida E (2008). Down-regulation of the PI3-kinase/Akt pathway by ERK MAP kinase in growth factor signaling. Genes Cells.

[13] Zhou Jing, Du Ting, Li Baoman, Rong Yan, Verkhratsky Alexei, Peng Liang (2015). Crosstalk Between MAPK/ERK and PI3K/AKT Signal Pathways During Brain Ischemia/Reperfusion. ASN Neuro.

[14] Ranganathan S, Bowser R (2003). Alterations in G(1) to S phase cell-cycle regulators during amyotrophic lateral sclerosis. Am J Pathol.

[15] Rodriguez J, Calvo F, Gonzalez JM, Casar B, Andres V, Crespo P (2010). ERK1/2 MAP kinases promote cell cycle entry by rapid, kinase-independent disruption of retinoblastoma-Lamin a complexes. J Cell Biol.

