# Supplementary material for: Loss of endosomal recycling factor RAB11 coupled with complex regulation of MAPK/ERK/AKT signaling in postmortem spinal cord specimens of sporadic amyotrophic lateral sclerosis patients
Source: Mol Brain. 2019 Jun 13;12:55. doi: 10.1186/s13041-019-0475-y (PMC6567394; doi:10.1186/s13041-019-0475-y)

Additional Files: Supplementary Figures

Loss of endosomal recycling factor RAB11 coupled with complex regulation of MAPK/ERK/AKT signaling in postmortem spinal cord specimens of sporadic amyotrophic lateral sclerosis patients

Joy Mitra[1], Pavana M. Hegde[1] and Muralidhar L. Hegde[1,2,3*]

[1]Department of Radiation Oncology, Houston Methodist Research Institute, Houston, TX 77030, USA; [2]Weill Medical College, New York, NY 10065, USA; [3]Houston Methodist Neurological Institute, Institute of Academic Medicine, Houston Methodist, Houston, TX 77030, USA

* Corresponding Author: Muralidhar L. Hegde (mlhegde@houstonmethodist.org)

Email addresses of authors:

Joy Mitra (jmitra@houstonmethodist.org)

Pavana M. Hegde (pdixit@houstonmethodist.org)

Muralidhar L. Hegde (mlhegde@houstonmethodist.org)

Supplementary Material included two Additional Figures.

Additional file 1: Figure S1. Relative levels of monomeric TDP-43 and RAB11 in ALS patients spinal cord tissue. Related to Fig. 1a. Histogram plot showing relative expression levels of monomeric TDP-43 and RAB11 in spinal cord tissue extracts from four controls and 10 ALS patients. X-axis indicates sample number and Y-axis denotes relative protein level, quantified from wester blots. The TDP-43 monomer levels were previously reported in Mitra et al. [8] and RAB11 levels are shown in Fig. 1a in this study.

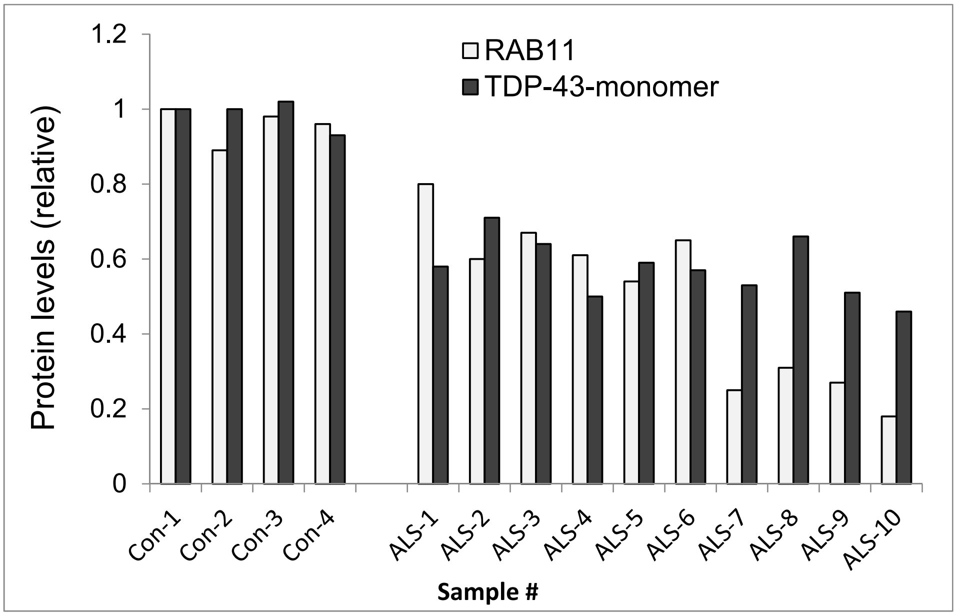

Supplement: Supplementary file 1 — Figure S1. Relative levels of monomeric TDP-43 and RAB11 in ALS patients spinal cord tissue. Related to Fig. 1a. Histogram plot showing relative expression levels of monomeric TDP-43 and RAB11 in spinal cord tissue extracts from four controls and 10 ALS patients. X-axis indicates sample number and Y-axis denotes relative protein level, quantified from western blots. The TDP-43 monomer levels were previously reported in Mitra et al. [8] and RAB11 levels are shown in Fig. 1a in this study. (PDF 481 kb) [file 13041_2019_475_MOESM1_ESM.pdf]
