# Supplementary material for: Loss of endosomal recycling factor RAB11 coupled with complex regulation of MAPK/ERK/AKT signaling in postmortem spinal cord specimens of sporadic amyotrophic lateral sclerosis patients
Source: Mol Brain. 2019 Jun 13;12:55. doi: 10.1186/s13041-019-0475-y (PMC6567394; doi:10.1186/s13041-019-0475-y)

Additional file 2: Figure S2. AKT/MAPK/ERK signaling in TDP-43-ALS patients. Related to Figure 1a. Representative western blot images of total spinal cord (post-mortem) tissue lysates from four controls and 10 ALS probed with anti-ERK1 (GTX100699), anti-ERK2 (GTX113094), anti-p90RSK (sc-74459), anti-AKT (sc-271149) and anti-GAPDH (NB-300-285) antibodies. GAPDH served as the loading control.

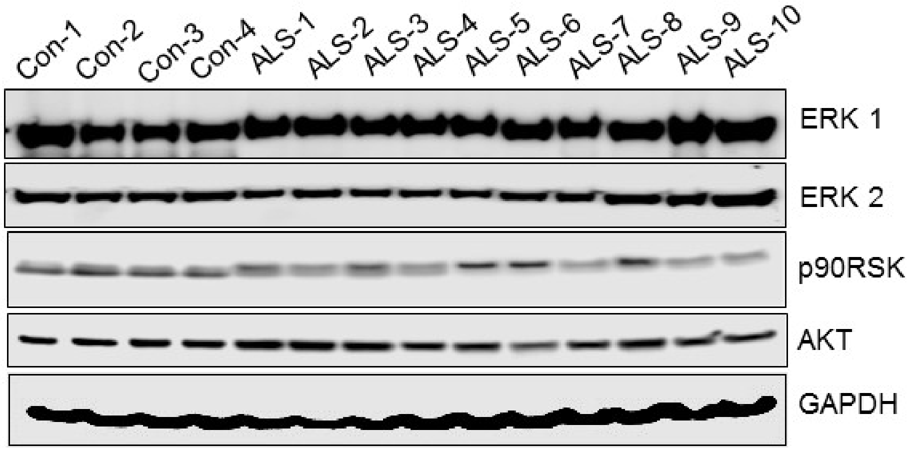

Supplement: Supplementary file 2 — Figure S2. AKT/MAPK/ERK signaling in TDP-43-ALS patients. Related to Fig. 1a. Representative western blot images of total spinal cord (post-mortem) tissue lysates from four controls and 10 ALS probed with anti-ERK1 (GTX100699), anti-ERK2 (GTX113094), anti-p90RSK (sc-74459), anti-AKT (sc-271149) and anti-GAPDH (NB-300-285) antibodies. GAPDH served as the loading control. (PDF 484 kb) [file 13041_2019_475_MOESM2_ESM.pdf]
